# Efficacy and Safety of Edoxaban in Cancer-Associated Venous Thromboembolism: A Real World Retrospective Study

**DOI:** 10.1055/a-1783-9744

**Published:** 2022-03-01

**Authors:** Elisa Grifoni, Andrea Baroncelli, Gabriele Pinto, Eleonora Cosentino, Irene Micheletti, Ira Signorini, Grazia Panigada, Giancarlo Landini, Luca Masotti

**Affiliations:** 1 Internal Medicine II, S. Giuseppe Hospital, Azienda USL Toscana Centro, Empoli, Italy; 2 Internal Medicine, SS. Cosma and Damiano Hospital, Azienda USL Toscana Centro, Pescia, Italy; 3 Internal Medicine, S. Maria Nuova Hospital, Azienda USL Toscana Centro, Florence, Italy

**Keywords:** cancer, venous thromboembolism, bleeding, edoxaban, low molecular weight heparin

## Abstract

**Introduction** Few data exist on the use of edoxaban in cancer-associated venous thromboembolism (VTE) outside of clinical trials. Aim of this study was to evaluate the characteristics and outcomes of these patients in a real world clinical setting.

**Methods** We retrospectively analyzed the characteristics of patients with cancer-associated VTE who were prescribed edoxaban. Follow-up at 3, 6, and 12 months was performed: VTE recurrences, bleedings, mortality, cancer progression and treatment, edoxaban interruption and its reason were assessed.

**Results** Fifty-four patients, 38 females (70.4%), mean age 71 ± 14 years, were enrolled. In 38 patients (70.4%), the episode of VTE was the first one, in 28 (51.8%) it was an isolated deep vein thrombosis (DVT), in 13 (24.1%) a pulmonary embolism (PE) associated with DVT, in 13 (24.1%) an isolated PE. Median time between cancer and VTE diagnosis was 6 (interquartile range [IQR] 2–47) months. Median time between VTE diagnosis and edoxaban prescription was 36 (IQR 7–117) days. At 3, 6, and 12 months the incidence of all-cause mortality was 16.6, 22.2, and 38.8%, that of VTE recurrence 1.8, 1.8, and 3.7%, and that of major bleeding 7.4, 9.2, and 12.9%, respectively. No bleeding was fatal. Of the 33 patients alive at 12 months, 32 (96.9%) were still on edoxaban therapy, in seven (21.2%) cancer was in progression.

**Conclusion** Our study, conducted on a real world population of patients with cancer-associated VTE, confirms the results of randomized controlled clinical trials, and supports the use of edoxaban as effective and safe treatment in this context.

## Introduction

In recent years direct oral anticoagulants (DOACs) have shown similar efficacy and better safety profile compared with vitamin K antagonists, becoming the standard of care for the treatment of venous thromboembolism (VTE) in the general population. In the main phase III randomized clinical trials on DOACs for VTE treatment, a small percentage of patients, around 5 to 6%, were affected by cancer. 1 Subsequent meta-analyses showed that DOACs could represent an alternative to conventional treatment in patients with cancer-associated VTE, since they seemed to be not inferior to low molecular weight heparin (LMWH) in terms of incidence of recurrent VTE and bleeding. 2 The phase III randomized controlled clinical trials HOKUSAI-VTE CANCER, 3 4 SELECT-D, 5 and the more recent ADAM-VTE 6 and CARAVAGGIO 7 confirmed the efficacy and safety of DOACs in patients with cancer-associated VTE. Based on the results of these trials, since 2018 international guidelines recommend the use of DOACs as treatment of choice for such patients together with LMWH. 8 9 10 11 12 However, few data exist on the use of DOACs in patients with cancer-associated VTE in the real world as well as on 12-month follow-up. 13

Aim of this study was to evaluate the clinical characteristics and outcomes of a cohort of patients with cancer-associated VTE treated with edoxaban outside of randomized controlled clinical trials.

## Materials and Methods

We retrospectively analyzed the demographic, clinical, and instrumental characteristics of patients treated with edoxaban for VTE, who were referred to our clinic from April 2017 to August 2019. Among these patients, those with history of active cancer were identified. Active cancer was defined as a cancer that had been diagnosed within the past 6 months, cancer for which anticancer treatment was being given at the time of or during the 6 months before edoxaban prescription, or recurrent locally advanced or metastatic cancer.

For all the patients, age at the time of VTE diagnosis, sex, date of cancer and VTE diagnosis, date of edoxaban prescription, type of VTE (deep vein thrombosis, DVT, superficial vein thrombosis, SVT, pulmonary embolism, PE), history of previous VTE, presence of non-oncological comorbidities, body weight, and blood tests at the time of edoxaban prescription were analyzed. In the case of DVT, site and side (lower or upper limbs, uni- or bilateral, proximal or distal) and the possible association with central venous catheters insertion were recorded. In the case of PE, the simplified Pulmonary Embolism Severity Index (sPESI) was calculated, and risk stratification was assessed according to the European Society of Cardiology (ESC) 2014 to 2019 Guidelines. 14 For the VTE index event, type of acute treatment and need for hospitalization were also reported. Data on type (solid tumor or hematological malignancy), primary site, cancer staging (locally advanced or metastatic), chemotherapy (type of drug/s and date of the last administration with respect to VTE diagnosis and edoxaban prescription), radiotherapy (date of the last treatment with respect to VTE diagnosis and edoxaban prescription), any surgical intervention, and need for palliative care activation were recorded.

For all the patients, a follow-up visit was performed at 3, 6, and 12 months. At each visit, recurrence of VTE, minor, clinically relevant non-major (CRNM) or major bleeding, according to the International Society on Thrombosis and Hemostasis (ISTH) definition, 15 VTE-related, cancer-related or any-cause mortality, cancer progression and treatment, possible edoxaban therapy interruption and its reason were assessed.

All procedures performed in this study were in accordance with the ethical standards of the institutional research committee and with the 1964 Helsinki declaration and its later amendments. All the enrolled patients consented to participate in the study.

### Statistical Analysis

Continuous variables were expressed as mean ± standard deviation (SD) or median and interquartile range (IQR), as appropriate. Categorical variables were presented as counts and percentages. Categorical variables were compared using Fisher's exact test. Survival curves were generated with the use of the Kaplan-Meier method, and the difference between groups was assessed by log-rank test. A *p* -value of <0.05 was considered statistically significant.

## Results

### Baseline Patients' Characteristics

The study population consisted of 54 patients, 38 females (70.4%), with the mean age of 71 ± 14 years. The median time between VTE and cancer diagnosis was 6 (IQR 2–47) months. The episode of VTE was an isolated DVT in 28 patients (51.8%), a PE associated with DVT or SVT in 13 cases (24.1%), an isolated PE in 13 (24.1%). DVT involved the upper limbs in five patients (12.2%), was bilateral in six cases (14.6%), and proximal in 33 (80.5%). The episode of VTE was asymptomatic in 11 patients with isolated PE (20.4%). In patients with PE the median sPESI score was 2 (IQR 1–2). The ESC risk was low-intermediate in 24 patients (92.3%), high-intermediate in two (7.7%). In five patients (9.2%) the episode of VTE was associated with the presence of a central venous catheter. Sixteen patients (29.6%) had a history of previous VTE. In 30 patients (55.5%) hospitalization was needed, in 23 of them (76.6%) VTE was the reason of admission. Table 1 summarizes the baseline characteristics of the study patients.

**Table 1:** Baseline characteristics of patients: all patients, and comparison between patients treated with enoxaparin/fondaparinux and those treated with edoxaban in the post-acute phase of cancer-associated VTE

	All ( *n* = 54)	E/F ( *n* = 26)	E ( *n* = 28)	*p* -Value
Mean age ± SD (years)	71 ± 14	73 ± 10	70 ± 12	0.325
Female sex, *n* (%)	38 (70.4)	17 (65.3%)	21 (75%)	0.554
Previous VTE, *n* (%): • Isolated DVT • Isolated PE • PE + DVT • SVT	16 (29.6) 11 (20.3) 2 (3.7) 2 (3.7) 1 (1.8)	6 (23.1) 5 (19.2) 0 (0) 1 (3.8) 0 (0)	10 (35.7) 6 (21.4) 2 (7.1) 1 (3.6) 1 (3.6)	0.379
Cardiovascular risk factors and comorbidities, *n* (%): • Arterial hypertension • Smoking habit • Diabetes mellitus • Chronic obstructive lung disease • HIV/HBV/HCV infection • Coronary artery disease • Atrial fibrillation	17 (31.5) 9 (16.7) 7 (12.9) 6 (11.1) 2 (3.7) 1 (1.8) 1 (1.8)	8 (30.8) 5 (19.2) 2 (7.7) 3 (11.5) 1 (3.8) 0 (0) 0 (0)	9 (32.1) 4 (14.3) 5 (17.9) 3 (10.7) 1 (3.6) 1 (3.6) 1 (3.6)	1.000 0.723 0.423 1.000 1.000 1.000 1.000
Body weight <60 kg, *n* (%):	14 (25.9)	7 (26.9)	7 (25)	1.000
Moderate renal failure (CrCl 30–50 mL/min), *n* (%):	9 (16.7)	4 (15.4)	5 (17.9)	1.000
Thrombocytopenia (Plt <100 × 10 ^9^ /L), *n* (%):	2 (3.7)	2 (7.7)	0 (0)	0.227
Solid tumor, *n* (%): • Lung • Genitourinary ○ Uterine/Ovarian ○ Kidney ○ Prostate • Breast • Colorectal • Pancreatic/Hepatobiliary • Gastric • Other: ○ Brain ○ Skin ○ Mascellar sinus	48 (88.8) 10 (18.5) 13 (24.1) 7 (7.4) 3 (5.5) 3 (5.5) 9 (16.7) 7 (12.9) 4 (7.4) 2 (3.7) 1 (1.8) 1 (1.8) 1 (1.8)	23 (88.5) 5 (19.2) 6 (23.1) 4 (15.4) 1 (3.8) 1 (3.8) 4 (15.4) 4 (15.4) 1 (3.8) 0 (0) 1 (3.8) 1 (3.8) 1 (3.8)	25 (89.3) 5 (17.9) 7 (25) 3 (10.7) 2 (7.1) 2 (7.1) 5 (17.9) 3 (10.7) 3 (10.7) 2 (7.1) 0 (0) 0 (0) 0 (0)	1.000
Hematological malignancy, *n* (%)	6 (11.2)	3 (11.5)	3 (10.7)	1.000
Metastatic tumor, *n* (%)	18 (37.5)	8 (30.8)	10 (35.7)	0.777

Abbreviations: CrCl, creatinine clearance; DVT, deep vein thrombosis; E, patients treated with edoxaban in the post-acute phase; E/F, patients treated with enoxaparin/fondaparinux in the post-acute phase; HBV, hepatitis B virus; HCV, hepatitis C virus; HIV, human immunodeficiency virus; IQR, interquartile range; PE, pulmonary embolism; Plt, platelets; SVT, superficial vein thrombosis; VTE, venous thromboembolism.

Forty-eight patients (88.8%) were affected by solid cancer (18 metastatic, 37.5%), six (11.2%) by hematological malignancy ( Table 1 ). Thirty-three patients (61.1%) had received chemotherapy in the previous 12 months [24 (44.4%) in the previous month, two (3.7%) within 3 and 6 months, seven (12.8) within 6 and 12 months before the VTE index event)]. Anticancer drug therapies used are reported in Supplementary Table S1 . Twenty-one patients (38.8%) had undergone radiotherapy in the previous 12 months [six (11.1%) in the previous month, one (1.8%) within 1 and 3 months, three (5.5%) within 3 and 6 months, 11 (20.4%) within 6 and 12 months before the VTE index event)]. Twenty-eight patients (51.8%) had undergone surgery; the median time between surgery and VTE occurrence was 20 (IQR 3.75–68) months. At the time of VTE diagnosis one patient (1.8%) was on palliative treatment.

### Acute and Post-Acute Phase VTE Treatment

Acute treatment of VTE at diagnosis was performed with fondaparinux in 32 patients (59.3%), enoxaparin in 20 (37%), and intravenous unfractionated heparin in two (3.7%). In the post-acute phase (7 days to 6 months), 28 patients (51.9%) were treated with edoxaban, and 26 (48.1%) with fondaparinux 7.5 mg once daily (20 patients, 37%) or enoxaparin 100 IU/kg twice daily (six patients, 11.1%). In the latter group, edoxaban was prescribed after the first 6 months of therapy with enoxaparin or fondaparinux. The median time from VTE diagnosis to edoxaban prescription was 36 (IQR 7–117) days. In patients undergoing treatment with edoxaban between 7 days and 6 months, edoxaban was started on average 11 ± 9 days after VTE diagnosis, in those treated with edoxaban after 6 months, edoxaban was started on average 136 ± 83 days after the VTE episode. In 32 patients (59.2%) edoxaban was prescribed at full dose, in 22 (40.8%) at reduced dose. In 11 of 22 patients (50%) the reason for reduced dose prescription was low body weight (<60 kg), in eight (36.5%) moderate renal failure, in three (13.5%) the presence of both conditions. During follow-up one patient required edoxaban dose adjustment (from reduced to full dose) due to improvement of renal function, whereas in one patient on low dose edoxaban the drug was temporarily discontinued due to acute renal failure.

No significant differences were found in terms of clinical characteristics (type of VTE, comorbidities, type of malignancy) between patients treated with enoxaparin or fondaparinux for the first 6 months and those who received edoxaban in the subacute phase (7 days to 6 months) ( Table 1 ).

### Outcomes

Tables 2a and 2b summarize outcomes at 3-, 6- and 12-month follow-up observed in all patients, and separately, in patients treated with enoxaparin/fondaparinux and those treated with edoxaban in the post-acute phase of cancer-associated VTE.

**Table 2:** Cumulative outcomes at 3-, 6- and 12-month follow-up: all patients ( Table 2a ), and comparison between patients treated with enoxaparin/fondaparinux and those treated with edoxaban in the post-acute phase of cancer-associated VTE ( Table 2b )

**a.**									
	**3 mo**			**6 mo**			**12 mo**		
Total mortality, *n* (%)	9 (16.6)			12 (22.2)			21 (38.8)		
Cancer-related mortality, *n* (%)	7 (12.9)			10 (18.5)			18 (33.3)		
VTE recurrences, *n* (%)	1 (1.8)			1 (1.8)			2 (3.7)		
Total bleedings, *n* (%)	4 (7.4)			5 (9.2)			8 (14.8)		
• Major bleedings, *n* (%)	4 (7.4)			5 (9.2)			7 (12.9)		
• CRNMBs, *n* (%)	0 (0)			0 (0)			1 (1.8)		
• Minor bleedings, *n* (%)	0 (0)			0 (0)			0 (0)		
**b.**									
	**3 mo**			**6 mo**			**12 mo**		
	**E/F (** ***n*** ** = 26)**	**E (** ***n*** ** = 28)**	***p*** **-Value**	**E/F (** ***n*** ** = 26)**	**E (** ***n*** ** = 28)**	***p*** **-Value**	**E/F (** ***n*** ** = 26)**	**E (** ***n*** ** = 28)**	***p*** **-Value**
Total mortality, *n* (%)	2 (7.7)	7 (25)	0.144	2 (7.7)	10 (35.7)	**0.021**	6 (23.1)	15 (53.6)	**0.028**
Cancer-related mortality, *n* (%)	1 (3.8)	6 (21.4)	0.102	1 (3.8)	9 (32.1)	**0.012**	5 (19.2)	13 (46.4)	**0.046**
VTE recurrences, *n* (%)	0 (0)	1 (3.6)	1.000	0 (0)	1 (3.6)	1.000	1 (3.8)	1 (3.6)	1.000
Total bleedings, *n* (%)	1 (3.8)	3 (10.7)	0.611	1 (3.8)	4 (14.3)	0.353	1 (3.8)	7 (25)	0.052
• Major bleedings, *n* (%)	1 (3.8)	3 (10.7)	0.611	1 (3.8)	4 (14.3)	0.353	1 (3.8)	6 (21.4)	0.102
• CRNMBs, *n* (%)	0 (0)	0 (0)	1.000	0 (0)	0 (0)	1.000	0 (0)	1 (3.6)	1.000
• Minor bleedings, *n* (%)	0 (0)	0 (0)	1.000	0 (0)	0 (0)	1.000	0 (0)	0 (0)	1.000

Abbreviations: CRNMB, clinically relevant non-major bleeding; E/F, patients treated with enoxaparin/fondaparinux in the post-acute phase; E, patients treated with edoxaban in the post-acute phase; VTE, venous thromboembolism.

At 90 days, nine patients (16.6%) had died, one (1.8%) patient had recurrent VTE (during edoxaban interruption for hemopericardium), and four patients (7.4%) had a bleeding complication; in seven of nine patients (12.9%) the cause of death was cancer-related, in two (3.7%) death was due to sepsis. At 6 months, 12 patients (22.2%) had died (cancer-related mortality 18.5%); no further recurrence of VTE had occurred, while the overall frequency of bleeding was 9.2%. At 12 months, 21 deaths (38.8%; cancer-related mortality 33.3%), two VTE recurrences (3.7%), and eight bleedings (14.8%) were recorded ( Table 2a ). Of the 33 survived patients, 32 (96.9%) were still on edoxaban therapy. In seven of 33 patients (21.2%) the neoplastic disease was in progression. In the only patient in whom therapy had been interrupted, the neoplastic disease was in complete remission.

**Fig. 1:**
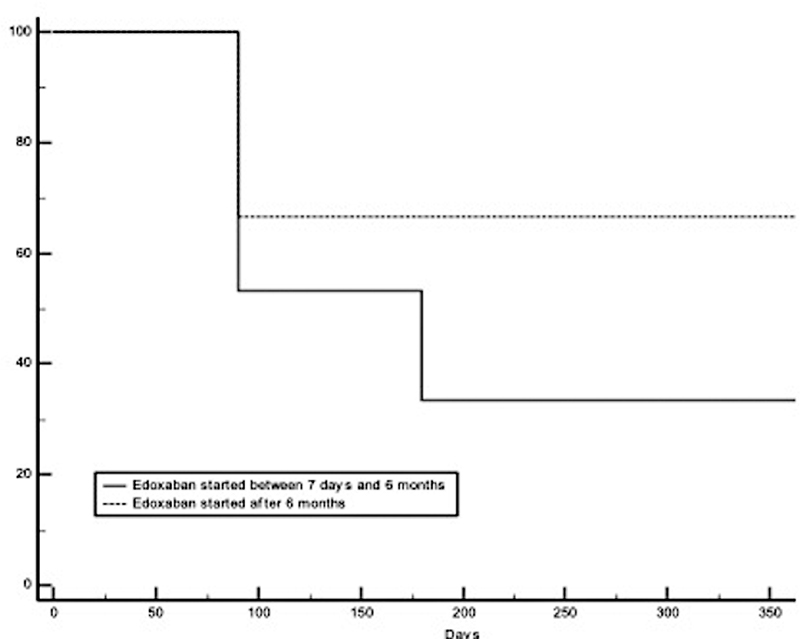
Twelve-month survival curves of patients treated with edoxaban between 7 days and 6 months and those treated with edoxaban after 6 months from cancer-associated VTE. VTE, venous thromboembolism.

Median survival was 365 (IQR 364.2–365) days. Total mortality and cancer-related mortality at 6 and 12 months were significantly higher in patients treated with edoxaban than in those treated with enoxaparin/fondaparinux in the post-acute phase of cancer-associated VTE ( Table 2b ). Fig. 1 shows 12-month survival curves of the two groups of patients.

**Fig. 2:**
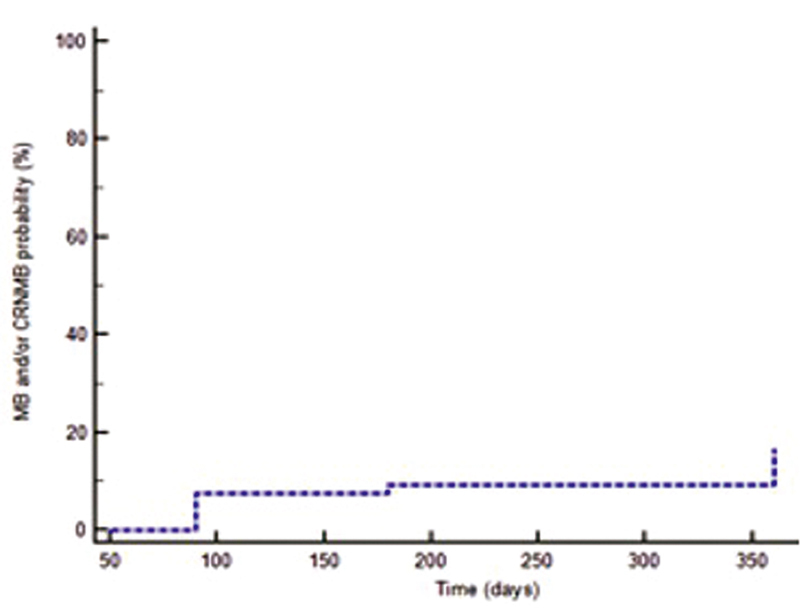
Timing and incidence of major and/or clinically relevant non-major bleedings at 12-month follow-up. CRNMB, clinically relevant non-major bleeding; MB, major bleeding.

Among eight bleeding events, seven (12.9%) were classified as major, one (1.8%) as CRNM; regarding the site of bleeding, three were gastrointestinal, two were represented by a fall in hemoglobin levels of 2 g/dL or more, one was pericardial, one retroperitoneal, and one an intraocular bleeding. In all these patients anticoagulant therapy was temporarily discontinued. No bleeding event was fatal. Four patients (7.4%) who had experienced bleeding died at 12-month follow-up (two at 90 days, one between 90 days and 6 months, and one between 6 and 12 months). In patients who experienced bleeding during treatment with edoxaban the tumor sites were: pancreas (two), stomach (one), liver (one), lung (one), breast (one), prostate (one), and kidney (one). Among the four gastrointestinal cancer patients who experienced bleeding (30.7%), two (50%) had a gastrointestinal bleeding; both of these latter patients had metastatic gastric cancer, and one had low hemoglobin levels prior to bleeding. In Fig. 2 , timing and incidence of major and/or CRNM bleedings at 12-month follow-up are shown. Median time to bleeding was 200 (IQR 110.7–316.2) days.

The difference in the incidence of bleeding and symptomatic VTE recurrence between patients treated with enoxaparin/fondaparinux and those treated with edoxaban in the post-acute phase of cancer-associated VTE was not statistically significant ( Table 2b ).

## Discussion

Our study was aimed at providing evidence from the real world, analyzing the characteristics and the 12-month outcomes of patients with cancer-associated VTE who were prescribed edoxaban in daily clinical practice, outside of clinical trials.

The phase III randomized controlled clinical trials on DOACs for cancer-associated VTE 3 4 5 6 7 showed their efficacy and safety also for the treatment of these patients, with the issue of gastrointestinal bleeding, which, however, was not increased in the studies with apixaban. In the HOKUSAI-VTE CANCER study the treatment duration was 12 months, while in the SELECT-D, ADAM-VTE and CARAVAGGIO studies it was 6 months. Cumulatively, in these four trials, in patients receiving DOACs the incidence of VTE recurrence was 5.2% (8.2% in the dalteparin arm, RRR 38%), that of major bleeding 4.3% (3.3% in the dalteparin arm, RRR 31%), and that of all-cause mortality 23.9% (24.2% in the dalteparin arm, RRR 1%).

There is paucity of data on the use of DOACs in patients with cancer-associated VTE in the real world, and almost all involve rivaroxaban. These studies, similarly to our, are mostly retrospective and do not have a control group. In 2019, a systematic review by Søgaard et al 16 of previously published observational studies with rivaroxaban for the treatment of cancer-associated VTE reported an incidence of recurrent VTE ranging from 1.0 to 8.9% at 3-month follow-up, and from 4.0 to 13.2% at 6-month follow-up, and an incidence of major bleeding ranging from 2.4 to 17% at 3-month follow-up, and from 1.9 to 6% at 6-month follow-up. Using an American database, Kohn et al 17 found an incidence of VTE recurrence of 4.0%, major bleeding of 2.7%, and all-cause mortality of 11.3% at 180 days among 949 cancer patients treated with rivaroxaban. In this study, the highest incidence of major bleeding in patients with gastrointestinal cancer (6.1%) was confirmed. Similar results were reported by Soff et al, 18 with a 6-month cumulative incidence of VTE recurrence of 4.2%, major bleeding of 2.2%, and all-cause mortality of 22.2% on approximately 1,100 cancer patients treated with rivaroxaban. It was of great interest that 73.3% of major bleeding events involved the gastrointestinal tract, without, however, a significant association with gastrointestinal tumors. Our study showed similar incidences of recurrent VTE, major bleeding, and mortality at 6 months with edoxaban in the real world, and provided data also on 12-month outcomes.

Compared with phase III randomized controlled clinical trials on DOACs for cancer-associated VTE, 3 4 5 6 7 in our study patients had a higher average age (71 vs. 64–67 years). The percentage of patients on chemotherapy (61.1%) and with metastatic cancer (37.5%) was lower in our study than in phase III clinical trials, while that of patients with hematological malignancies (11.2%), previous VTE (29.6%) and moderate renal insufficiency (16.7%) were higher in our study; the percentage of patients with thrombocytopenia (3.7%) was similar ( Table 3 ). In our study the 6-month incidence of symptomatic recurrent VTE, the composite end point major and CRNM bleeding, and all-cause mortality were lower than those observed in phase III randomized controlled clinical trials, with the exception of the ADAM-VTE trial. It is of great interest to compare the 12-month outcomes observed in our study to those in the HOKUSAI-VTE CANCER study. In that trial, the 12-month incidence of symptomatic recurrent VTE (7.9%) and the combined end point major and/or CRNM bleeding (18.6%) were higher than in our study, with comparable rates of all-cause mortality (39.5%, of which 34.7% cancer-related). In a post hoc analysis of the HOKUSAI-VTE CANCER study focused on the follow-up period from 6 to 12 months, recurrent VTE occurred in two patients (0.7%) in the edoxaban group and in three patients (1.1%) in the dalteparin group, whereas major bleeding occurred in five (1.7%) and three patients (1.1%), respectively, so suggesting relatively low rates of recurrent VTE and major bleeding among patients with active cancer receiving extended anticoagulant therapy beyond 6 months. 19 In our study one VTE recurrence (1.8%) and two major bleedings (3.7%) were recorded during treatment with edoxaban between 6 and 12 months. It is also important to underline that the percentage of patients still on edoxaban treatment at 12-month follow-up was 59.2% in our study versus 38.3% (29.4% in the dalteparin arm) in the HOKUSAI-VTE CANCER study. 3

**Table 3:** Comparison of patients characteristics and 6-month outcomes between our study and the main randomized controlled clinical trials of DOACs for treatment of cancer-associated VTE

	HOKUSAI-VTE Cancer 3 4 (Edoxaban)	SELECT-D 5 (Rivaroxaban)	ADAM-VTE 6 (Apixaban)	CARAVAGGIO 7 (Apixaban)	Our study (Edoxaban)
Study design	RCT	RCT	RCT	RCT	Retrospective
Hematological malignancy, %	10.7	3	8.2	5.7	11.2
Metastatic cancer, %	52.5	58	65.3	67.5	37.5
Chemotherapy, %	71.6 b	85 d	73.5	85.6 c	61.1 a
Radiotherapy, %	NR	7	NR	NR	38.8 a
Previous VTE, %	9.4	NR	5.4	7.8	29.6
Body weight <60 kg, %	15.9	NR	12.9	NR	25.9
Moderate renal failure (CrCl 30–50 mL/min), %	7.3	NR	9.3	8.9	16.7
Thrombocytopenia (Plt <100 × 10 ^9^ /L), %	6.1	NR	6.7	3.6	3.7
DOAC reduced dose, %	23.4	NR	NR	NR	40.8
Alternative treatment, type: • Dalteparin, % • Enoxaparin, % • Fondaparinux, %	50.1 e – –	50 e – –	49.5 e – –	50.1 e – –	– 11.1 e 37 e
VTE recurrence, %	6.5	3.9	0.7	5.6	1.8
Major bleedings, %	5.6	5.4	0	3.8	9.2
CRNMB, %	12.3	12.3	6.2	9	0
Major bleedings + CRNMB, %	15.9	17.7	6.2	12.8	9.2
All-cause mortality, %	26.8	23.6	16	23.4	22.2

Abbreviations: CrCl, creatinine clearance; CRNMB, clinically relevant non-major bleeding.; DOAC, direct oral anticoagulant; NR, not reported; Plt, platelets; RCT, randomized controlled clinical trial; VTE, venous thromboembolism.

a In the last 12 mo.

b In the month before.

c At enrollment and/or in the previous 6 mo.

d At enrollment.

e Dalteparin 200 IU/kg once daily for the first 30 d, then 150 IU/kg once daily; Enoxaparin 100 IU/kg twice daily; Fondaparinux 7.5 mg once daily.

Therefore, our real world study seems to confirm the results of phase III randomized clinical trials in such patients, showing the efficacy of DOACs for the prevention of recurrent VTE and highlighting the risk of major or CRNM bleedings, which, however, do not seem to have higher incidences than those reported in the literature (even for LMWH or fondaparinux). The risk of bleeding, especially from gastrointestinal and genitourinary tracts, represents the main safety issue for the use of DOACs in patients with cancer-associated VTE. However, this is also an issue for non-cancer patients undergoing treatment with DOACs. A meta-analysis by Giustozzi et al 20 showed that, overall, in the four phase III randomized clinical trials (HOKUSAI-VTE CANCER, SELECT-D, ADAM-VTE, CARAVAGGIO) treatment with DOACs increases the risk of gastrointestinal bleeding by two times, even if not significant (RR 1.91, 95% CI: 0.96–3.82), while it significantly increases the risk of genitourinary bleeding by five times (RR 4.99, 95% CI: 1.08–23.08). However, it is still under investigation whether gastrointestinal and/or genitourinary bleeding is more frequent in patients with gastrointestinal and/or genitourinary tumors, since this type of bleeding also occurs in patients with neoplasm of different sites. In the HOKUSAI-VTE CANCER study, the percentage of patients with gastrointestinal cancer was 22.2%, and that of patients with genitourinary cancer was 12.5%. In that study, the incidence of major bleeding in patients with gastrointestinal cancer was significantly higher than in those with other site malignancies, and 91% of major bleedings in patients with gastrointestinal cancer treated with edoxaban occurred in the gastrointestinal tract (47% upper gastrointestinal tract). In patients with gastrointestinal cancer the incidence of gastrointestinal bleeding was 12.7% in the edoxaban arm and 3.6% in the dalteparin arm. The incidence of major bleeding in patients with genitourinary, pulmonary, breast and hematological malignancies treated with edoxaban was 4.6, 2.6, 0, and 1.8%, respectively. 3 4 A subsequent analysis of the HOKUSAI-VTE CANCER study on the efficacy and safety of edoxaban in patient groups with different types of cancer showed that edoxaban has a similar risk-benefit ratio to dalteparin in most cancer groups, but in those with gastrointestinal cancer the lower risk of recurrent VTE needs to be balanced against the increased risk of major bleeding. 21 In our study, approximately 24% of patients treated with edoxaban had a gastrointestinal tumor, and half of total bleedings occurred in these patients, being of gastrointestinal origin in 50% of cases. Recently, Bosch et al, in a nested case–control study in patients with gastrointestinal cancer randomized to edoxaban in the Hokusai VTE Cancer study, found that advanced cancer and low hemoglobin levels were associated with an increased risk of gastrointestinal bleeding in patients with gastrointestinal cancer receiving edoxaban. 22 In the SELECT-D study, in 80.5% of cases of major or CRNM bleedings, the site of bleeding was gastrointestinal or genitourinary. 5 In the CARAVAGGIO study, 32.6% of tumors were gastrointestinal and 11.5% genitourinary; although not exceeding bleedings in the dalteparin arm, 29.7% of major and/or CRNM bleedings with apixaban involved the gastrointestinal, and 31% the genitourinary tract. A subsequent analysis of the CARAVAGGIO study showed that major bleedings appear to be more common in patients with genitourinary and gastrointestinal cancer, but the rates of major bleeding in patients treated with apixaban or dalteparin were similar across patients with different cancer sites. 23

Therefore, it is evident that the use of DOACs in patients with cancer-associated VTE has some limitations, mainly represented by the risk of gastrointestinal and genitourinary bleeding. In addition, another limitation is represented by the type of anticancer therapy used, for the possible drug–drug interactions at the level of P-glycoprotein and/or cytochrome P450 3A4.

The selection of patients is therefore of main importance for the choice of anticoagulant treatment. Guidelines recommend avoiding the use of DOACs in patients with cancer and high risk of bleeding, such in those with gastrointestinal and genitourinary tumors, and in those with potential drug–drug interactions, which may cause dangerous increases or decreases in the concentration of the anticoagulant drug. 24 Personalization of therapy is therefore essential in patients with cancer-associated VTE. Although nowadays DOACs represent an effective and safe option at least in certain types of cancer, there are clinical situations and/or types of cancers, in which treating patients with LMWH or fondaparinux is still necessary. Finally, the decision on the type of treatment in cancer-associated VTE should take into account patient prognosis, with the awareness that, sometimes, the occurring of VTE may represent a negative prognostic factor. Therefore, in end-of-life patients with life expectancy of less than 3 months who develop VTE, it may be ethical to abstain from anticoagulant therapy and activate palliative care, as suggested by Kim et al. 25

Our study has some limitations, mainly due to the retrospective and single center design, the relatively small number of patients enrolled, and the lack of a control group. Moreover, almost half of the patients continued parenteral therapy for 6 months before switching to edoxaban introducing a possible selection bias in the study population, even if no significant differences were found between the two groups. However, we believe that our study may add an interesting contribution to the knowledge of the efficacy and safety of DOACs in patients with cancer-associated VTE in the real world, in particular providing evidence on the use of edoxaban in daily clinical practice.

## Conclusion

In conclusion, cancer-associated VTE represents one of the greatest challenges in the management of cancer patients. Nowadays, DOACs seem to be an effective and relatively safe therapeutic option. Our study, conducted on real world cancer-associated VTE patients treated with edoxaban, confirms the results of the HOKUSAI-VTE CANCER trial, with a rate of recurrent VTE of 3.7% and of major and/or CRNM bleedings of 14.8% at 12 months. Therefore, our study supports the use of edoxaban as effective and safe treatment in cancer-associated VTE patients without high risk of bleeding.
