# Supplementary material for: Efficacy and Safety of Edoxaban in Cancer-Associated Venous Thromboembolism: A Real World Retrospective Study
Source: TH Open. 2022 Mar 1;6(2):e99–e106. doi: 10.1055/a-1783-9744 (PMC9113858; doi:10.1055/a-1783-9744)
Supplement: Supplementary file 1 — Supplementary Material [file 10-1055-a-1783-9744-s210086.pdf]

Supplementary Table S1 Anticancer drug therapies

Antimetabolites, *n* (%):	9 (16.7)
Capecitabine	6 (11.1)
5-fluorouracil	1 (1.8)
Gemcitabine	1 (1.8)
Pemetrexed	1 (1.8)
Monoclonal antibodies, *n* (%):	7 (12.9)
Rituximab	3 (5.5)
Bevacizumab	1 (1.8)
Trastuzumab	1 (1.8)
Nivolumab	1 (1.8)
Pembrolizumab	1 (1.8)
Platinum-based agents, *n* (%):	6 (11.2)
Carboplatinum	4 (7.4)
Oxaliplatinum	2 (3.7)
Hormonal therapy, *n* (%):	5 (9.3)
Leuproreline	1 (1.8)
Abiraterone	1 (1.8)
Exemestane	1 (1.8)
Letrozole	1 (1.8)
Fulvestrant	1 (1.8)
Taxanes, *n* (%):	5 (9.3)
Paclitaxel	3 (5.5)
Docetaxel	2 (3.7)
Alkylating agents, *n* (%):	3 (5.5)
Cyclophosphamide	2 (3.7)
Bendamustine	1 (1.8)
Kinase inhibitors, *n* (%)	3 (5.5)
Gefitinib	1 (1.8)
Ibrutinib	1 (1.8)
Palbociclib	1 (1.8)
Vinca alkaloids, *n* (%):	3 (5.5)
Vincristine	2 (3.7)
Vinorelbine	1 (1.8)
Camptothecin, *n* (%):	2 (3.7)
Irinotecan	1 (1.8)
Topotecan	1 (1.8)
Immunomodulating agents, *n* (%):	2 (3.7)
Everolimus	1 (1.8)
Lenalidomide	1 (1.8)
Topoisomerase II inhibitors, *n* (%):	2 (3.7)
Etoposide	2 (3.7)
Alicondrine B analogues, *n* (%)	1 (1.8)
Eribulin	1 (1.8)
Other, *n* (%):	1 (1.8)
Idrossicarbamide	1 (1.8)

 © 2022. The Author(s).
